# Effect of additional body weight on arch index and dynamic plantar pressure distribution during walking and gait termination

**DOI:** 10.7717/peerj.8998

**Published:** 2020-04-21

**Authors:** Xuanzhen Cen, Datao Xu, Julien S. Baker, Yaodong Gu

**Affiliations:** 1 Faculty of Sports Science, Ningbo University, Ningbo, China; 2 Department of Sport and Physical Education, Hong Kong Baptist University, Hong Kong, China

**Keywords:** Arch height, Body loading, Plantar loading, Gait termination, Foot morphology

## Abstract

The medial longitudinal arch is considered as an essential feature which distinguishes humans from other primates. The longitudinal arch plays a supporting and buffering role in human daily physical activities. However, bad movement patterns could lead to deformation of arch morphology, resulting in foot injuries. The authors aimed to investigate any alterations in static and dynamic arch index following different weight bearings. A further aim was to analyze any changes in plantar pressure distribution characteristics on gait during walking and stopping, Twelve males were required to complete foot morphology scans and three types of gait tests with 0%, 10%, 20% and 30% of additional body weight. The dynamic gait tests included walking, planned and unplanned gait termination. Foot morphology details and plantar pressure data were collected from subjects using the Easy-Foot-Scan and Footscan pressure platform. No significant differences were observed in static arch index when adding low levels of additional body weight (10%). There were no significant changes observed in dynamic arch index when loads were added in the range of 20% to 30%, except in unplanned gait termination. Significant maximal pressure increases were observed in the rearfoot during walking and in both the forefoot and rearfoot during planned gait termination. In addition, significant maximum pressure increases were shown in the lateral forefoot and midfoot during unplanned gait termination when weight was increased. Findings from the study indicated that excessive weight bearing could lead to a collapse of the arch structure and, therefore, increases in plantar loading. This may result in foot injuries, especially during unplanned gait termination.

## Introduction

The medial longitudinal arch is the elastic and constrictive cambered structure in the foot, which has functions to support and maintain body balance during normal walking or with additional added weight (Zhao et al., 2018a; Zhao et al., 2018b). Foot morphology may change as a result of many factors, e.g., age, body weight, injury and type of footwear (Zhang, Li & Zhang, 2017). These morphological deformations might lead to a series of negative biomechanical effects (Zhang, Li & Zhang, 2017; Zhao et al., 2018a; Zhao et al., 2018b). Previous studies (Yan et al., 2013; Riddiford-Harland, Steele & Baur, 2010; Faria et al., 2010; Kitaoka, Luo & An, 1997; Bjelopetrovich & Barrios, 2016) have indicated that arch shape can be affected by increased body weight including obesity and excessive weight bearing. Riddiford-Harland, Steele & Baur (2010) compared differences in foot structure characteristics between obese and non-obese children and found that obese children tended to have fatter and flatter feet. Faria et al. (2010) also observed that obesity could lead to the collapse of the medial longitudinal arch as a result of excessive downward vertical forces. Additionally, Kitaoka, Luo & An (1997) observed that the foot arch would be flattened or deformed mildly by applying additional loads to the human foot. Alteration of the medial longitudinal arch has been reported with increased loading using an adjustable weighted vest (Bjelopetrovich & Barrios, 2016). The reduction in arch height exhibited linear and curvilinear trends with incremental loading (10% to 120% of additional body weight).

Excessive collapse of the medial longitudinal arch will lead to the loss of static and dynamic support function (Richie, 2007). Moreover, gait parameters of patients with collapsed arch’s may be affected with problems such as lower stride length, step width and speed (Shin et al., 2019). In addition to increased contact area in the medial midfoot, peak pressure and force was found to significantly decrease in the lateral forefoot in the flat foot type (Chuckpaiwong et al., 2008). However, a complete gait cycle also includes gait initiation and termination. Gait initiation and termination have been defined as mutual transitions between quiet standing posture and dynamic steady state walking, respectively (Yiou et al., 2017; Sparrow & Tirosh, 2005). Gait termination is a huge stability challenge for elderly individuals and patients with balance disorders, especially during unplanned gait termination (Sparrow & Tirosh, 2005). By comparing plantar pressure differences between planned gait termination (PGT) and unplanned gait termination (UGT), plantar pressure demonstrated an increased trend during UGT. Although plantar pressure increased in each anatomical region, there was no significant change in the midfoot contact area (Cen, Jiang & Gu, 2019). However, the biomechanical mechanism of gait termination in patients with flatfoot is unclear.

Studies related to arch characteristics for different body weights have mainly focused on cross-sectional experiments (Riddiford-Harland, Steele & Baur, 2010; Faria et al., 2010; Jankowicz-Szymańska et al., 2018). The main reason for this may be due to the subjects involved, and experimental conditions associated with longitudinal studies are difficult to control. As a result, there is a disparity between studies when researchers comparatively analyzed samples from previous studies. The differences observed were mainly derived from individual gait characteristics and arch types (Hotfiel et al., 2017). Previous researchers have attempted to imitate additional body weight and obesity by increasing external body loading (Vela et al., 1998). This methodology may assist researchers in understanding arch morphology at different body weights for further biomechanical studies.

The arch height index (AHI) was investigated by Williams and Mcclay in 2000. Since then AHI has been considered as a valid and reliable indicator for evaluating static arch features (Williams & Mcclay, 2000; Zhao et al., 2017; Liang et al., 2019). However, AHI is obtained by collecting the foot parameters (instep height and ball of foot length) from individuals under static conditions, and as a result, the arch behavior under dynamic loading may not be revealed (Bjelopetrovich & Barrios, 2016). For evaluating dynamic arch features, the dynamic arch index (AI), the ratio of contact area of the midfoot to the whole foot during movement, was evaluated and has been widely used since (Cavanagh & Rodgers, 1987; Yan et al., 2013).

The purpose of this study was to investigate any alteration in static and dynamic arch index and plantar pressure distribution characteristics using different added percentages of body weight (0%, 10%, 20% and 30% BW loads) during walking, planned and unplanned gait termination. A further aim was to explore deformational mechanism and biomechanical characteristic alterations of the foot arch under additional body weight conditions and provide insights into the prevention of injury. It was hypothesized that arch height would show reductions with body weight increments under both under static and dynamic conditions, and that maximal plantar pressure would increase as loads were added during walking and gait termination.

## Methods

### Subjects

Twelve subjects were recruited to participate in the study, with ages of 24.3  ± 0.6 years, heights of 175.5  ± 2.94 cm, weights of 68.0  ± 5.1 kg and foot lengths of 252.5  ± 1.4 mm. The key inclusion criteria were, (i) physically active male adults; (ii) the right leg as dominant; (iii) no hearing disorder; (iv) no disorders or injuries to the lower limbs in the first half of the year. All subjects were college students and had a history of running or other physical activities. None participated in experienced professional athletic training. Subjects were screened by a physician prior to experimentation and understood the purpose and procedure of the study. All subjects were fully familiarized with testing procedures prior to experimental data collection. The Ethics Committee of Ningbo University (RAGH20181218) approved the study and written informed consent was obtained from all individuals prior to participation.

### Procedures

**Figure 1:**
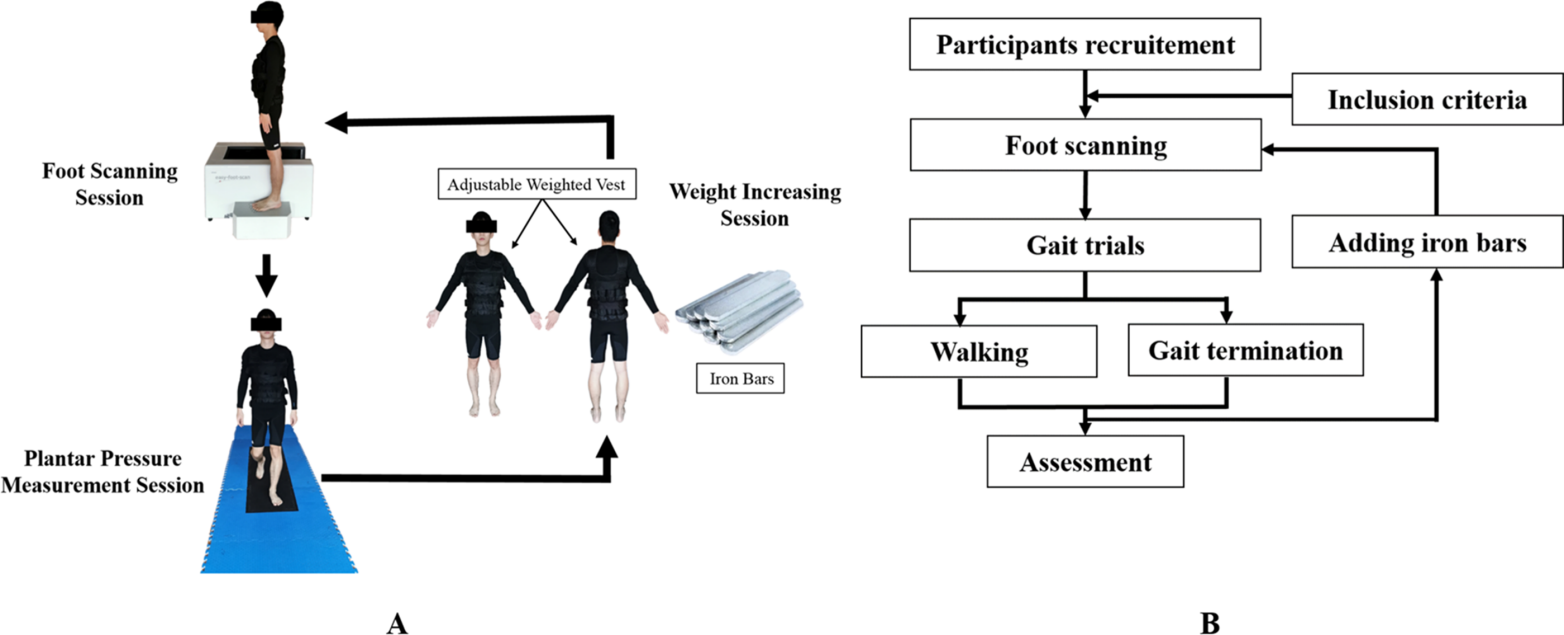
Protocol of foot scanning, plantar pressure collection and weight increasing. (A) Schematic view of foot scanning, plantar pressure collection and weight increasing; (B) flow chart of the procedures.

Individual subject weight was increased by wearing an adjustable weighted vest. The weight was controlled by adding calibrated iron bars to pockets in the vest, while keeping the center of subject mass unaffected. Based on their initial body weight (BW), individual percentage loaded increases in weight was designed to increase body mass by 0%, 10%, 20% and 30% BW. Subjects were also asked to continuously complete static foot morphology scans and gait trials on three occasions with increases in weight (increasing 10% BW each time) (Fig. 1). The dynamic gait trials included normal walking, PGT and UGT. The left lower limb was used for all experimental data collection.

Prior to the gait tests 3-Dimensional foot morphology images of all subjects with the addition of extra weight were collected using the Easy-Foot-Scan (OrthoBaltic, Kaunas, Lithuania). Compared to magnetic resonance imaging (MRI), 3-Dimensional foot morphological scanning has the advantages of quickness, convenience and cheapness, and has been proven previously to have superior reliability (Liang et al., 2019; Shu et al., 2015). The resolution, smoothing and hole filling were set at 1.0 mm, 30 mm and 100 mm, respectively (Cen et al., 2020). While the feet were scanned subjects were asked to stand barefoot in a standardized position while keeping the body mass stable without movement.

Three types of gait trials were performed along an 8m walkway, containing 2m Footscan pressure plate (RSscan International, Olen, Belgium) with a sampling rate of 250 Hz. Each subject was given five minutes to gain experience about the laboratory environment and to warm up. Firstly, subjects were asked to walk at their individual normal comfortable pace to present natural gait characteristics on the walkway while looking straight ahead.

**Figure 2:**
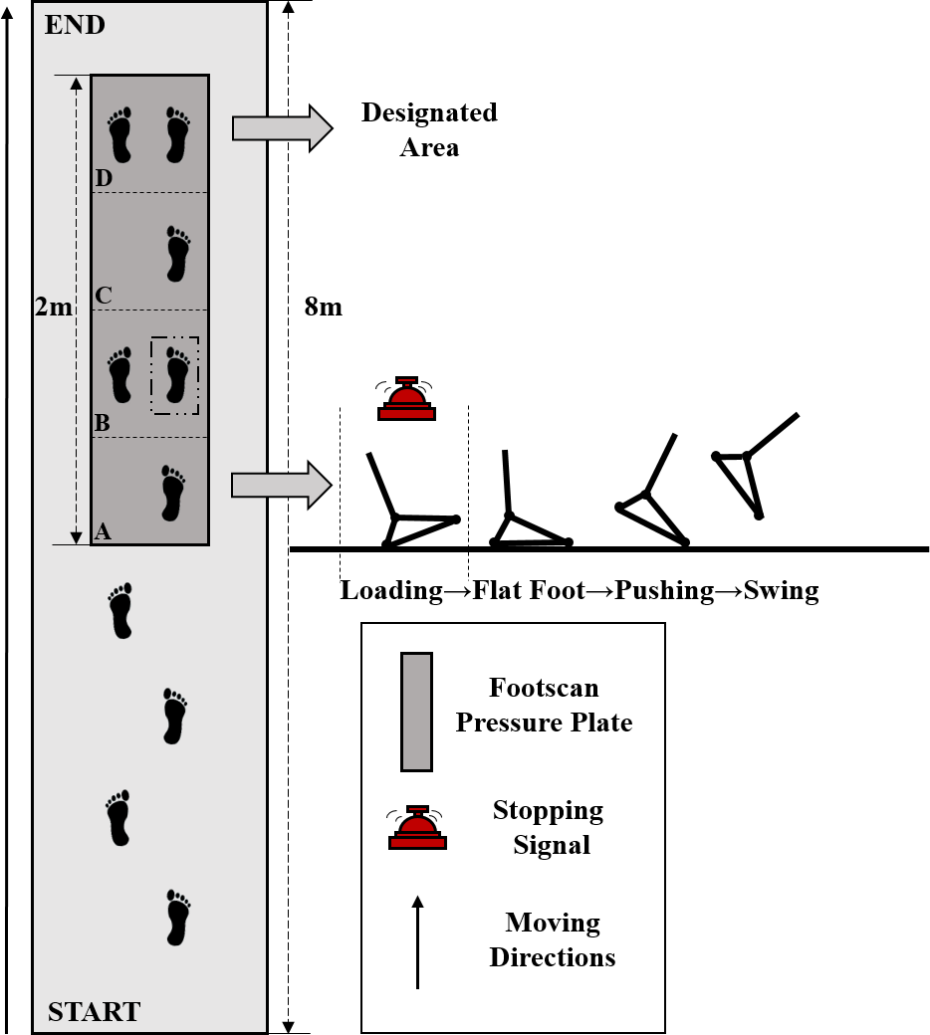
Overhead view of walkway used for gait experiments.

Subjects performed two types of gait termination tests on the walkway. The two-meter-long plantar pressure plate was artificially divided into four sections (A, B, C and D), and every section was about 50 cm*50 cm. Their primary task was to use the dominant leg (right leg) and the non-dominant leg (left leg) to pass section A and B respectively, and finally terminate the movement on a designated area (section D). Subjects received the termination signal as the heel of the right foot touched section A. Unplanned gait termination needed be executed to stop quickly on section B (Fig. 2). Twenty-five percent of tests in each block included a termination signal while the remaining 75% did not include a termination signal. In order to minimize experimental error, bell ringing in all trials was performed by the same experimenter. There was a two-minute rest interval between both trials to avoid fatigue affecting the accuracy of the experiment. Fifteen successful gait experimental data sets, including 5 walking trials, 5 PGT trials and 5 UGT trials, were required from each subject.

### Data acquisition

**Figure 3:**
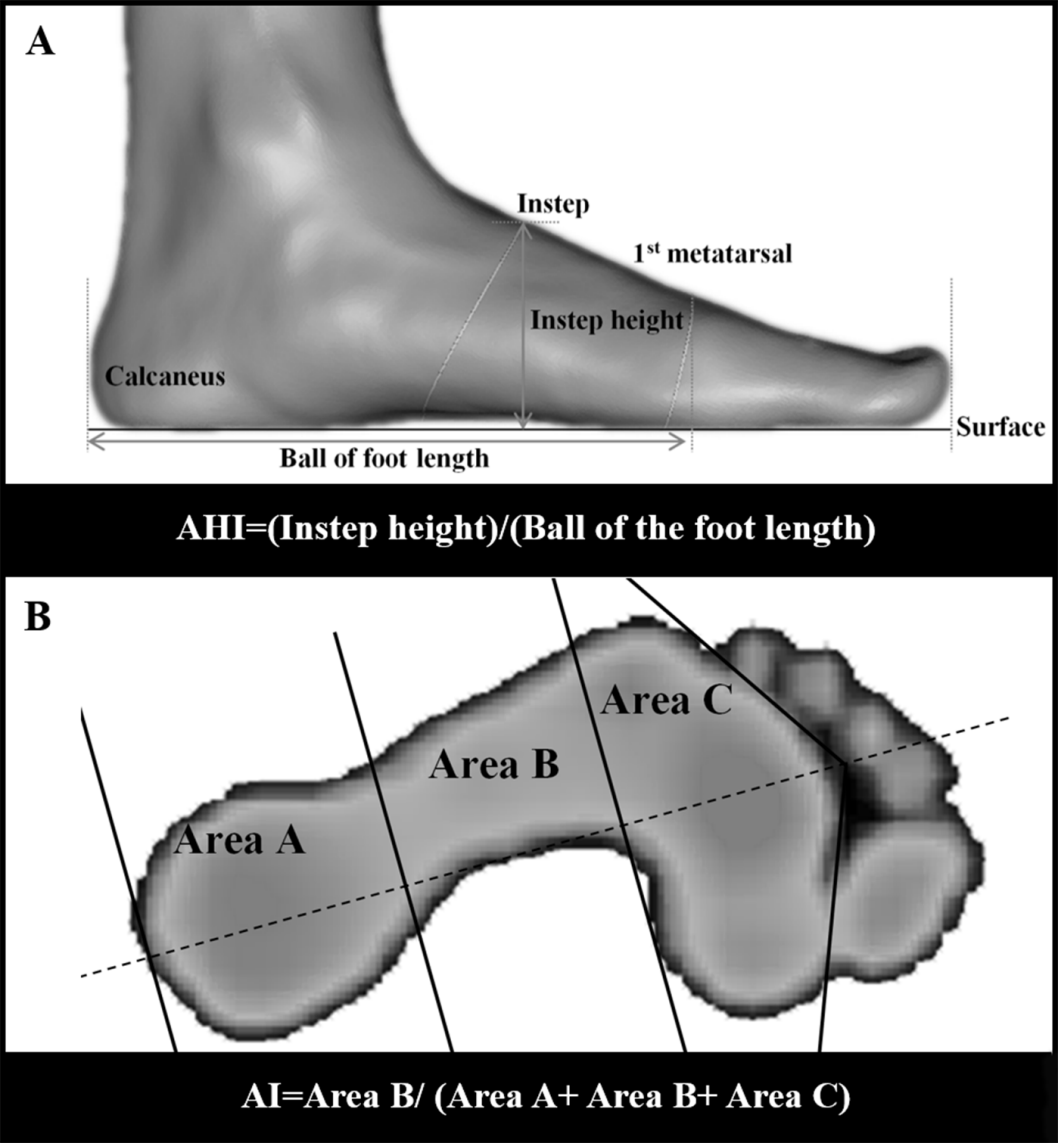
Measurements of AHI and AI. (A) The side view of three-dimensional foot morphology; (B) three parts of footprint without all toes: rearfoot, midfoot and forefoot.

Based on the two-dimensional foot image obtained from the foot morphological scanning, the variables of foot structure were calculated with Auto CAD software (Autodesk, San Rafael, CA, USA). Each subject’s AHI, as static arch index, was measured by the vertical distance from the instep point to the surface (instep height) divided by the distance from the first metatarsal joint protrusion to the most posterior point of the calcaneus (ball of the foot length) (Liang et al., 2019; Williams & Mcclay, 2000; Zhao et al., 2017) (Fig. 3A).

A foot axis line was drawn from the middle of the second metatarsal and third metatarsal to the middle of the calcaneus. Perpendicular to the foot axis line, the foot was divided in three equal parts: rearfoot (A), midfoot (B) and forefoot (C). The AI was defined as the ratio of the contact area between the midfoot and full foot without all toes (Cavanagh & Rodgers, 1987; Yan et al., 2013), and was measured using the following calculation: AI = Area B/(Area A + Area B + Area C) (Fig. 3B).

Based on the clinical relevance of increased plantar pressure, maximal pressure was selected among the plantar pressure indices (impulse, maximal force, contact area, load rate etc.) as a representative parameter to evaluate the plantar pressure distribution characteristics during walking and gait termination experiments using different loads (Hotfiel et al., 2017). The plantar surface was divided into three anatomical parts using the Footscan® 7.0 (RsScan International, Olen, Belgium), including rearfoot, midfoot and forefoot. The forefoot region included the first metatarsal (M1), second metatarsals (M2), third metatarsal (M3), fourth metatarsal (M4) and fifth metatarsal (M5). The rearfoot region included the medial heel (HM) and lateral heel (HL). The midfoot region only included the midfoot (MF). The plantar pressure data was normalized via dividing by the subjects’ initial body weight.

### Statistical analysis

Statistical analyses were performed with SPSS 19.0 for Windows™ software (IBM, Armonk, NY, USA). The Shapiro–Wilks test was applied to check that each variable was normally distributed. A repeated-measures ANOVA with Bonferroni adjustments was used to quantify the effects of the weight bearing conditions (0%, 10%, 20% and 30% BW additional loading) on each variable of the AHI, AI and maximal pressure in each anatomical region. In addition, AI and maximum pressure included data recorded during three types of gait trials (walking, PGT, and UGT), while AHI only included data from the subjects under static conditions. Bonferroni post-hoc pairwise comparisons were performed to provide details of significant differences for the four different weight bearing conditions. The observed Effect size (_*p*_*η*2) was also reported in this study. Data were illustrated as means and standard deviations (SD). The significance level was set at *p* < 0.05.

## Results

### Arch index

**Figure 4:**
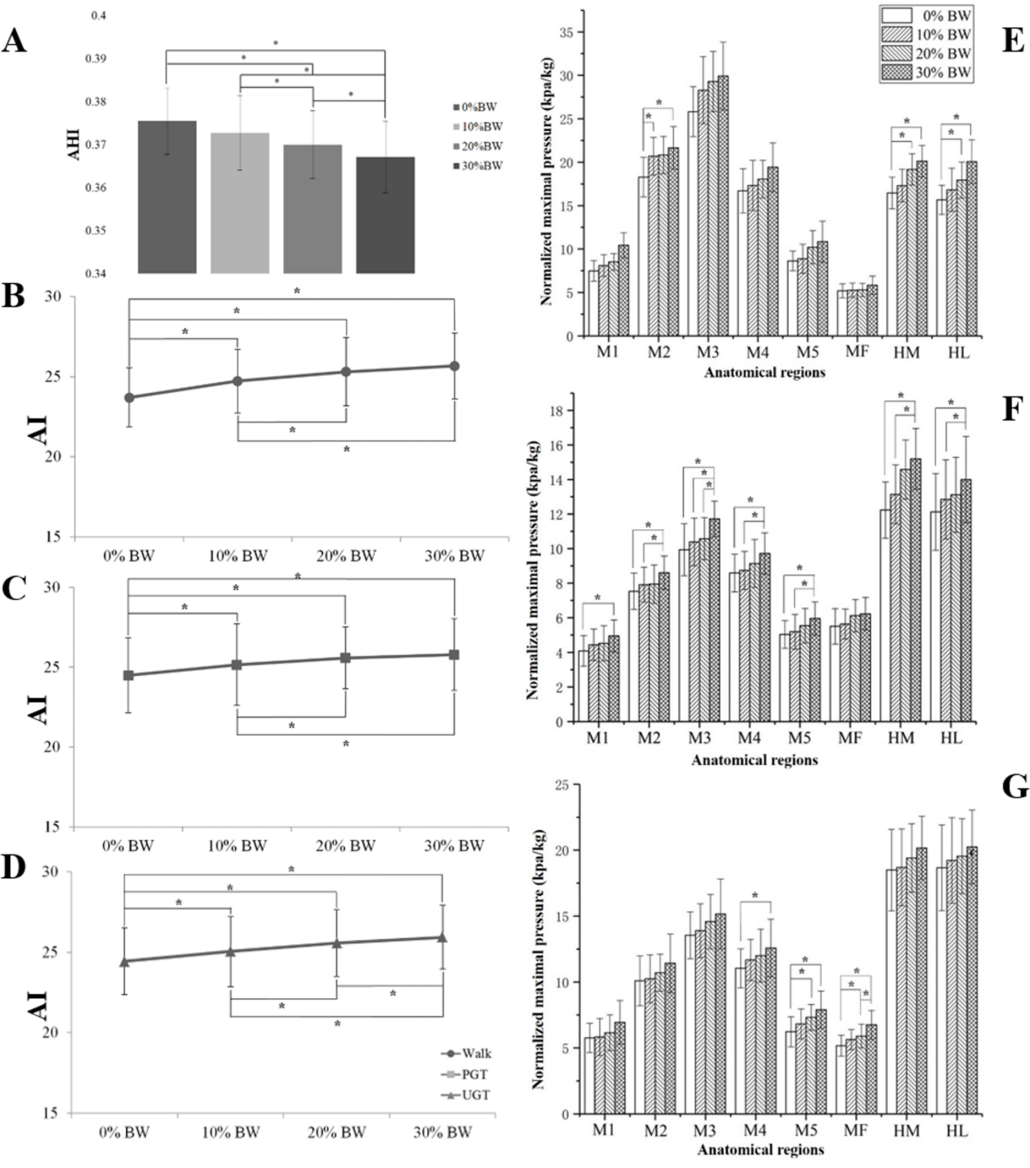
Comparisons of AHI, AI and plantar pressure in different additional body weight. (A) Comparisons of AHI in different additional body weight; (B) comparisons of AHI in different additional body weight during walking; (C) comparisons of AHI in different additional body weight during PGT; (D) comparisons of AHI in different additional body weight during UGT; (E) comparisons of maximal pressure in different additional body weight during walking; (F) comparisons of maximal pressure in different additional body weight during PGT; (G) comparisons of maximal pressure in different additional body weight during UGT; The symbol of ”*” represents a significant difference (*P* < 0.05).

Table 1, Figure 4A and Data S1 illustrate the effects of different loads for subject’s AHI. No significant differences were observed for 10% BW compared to the normal weight condition (*p* = 0.068 > 0.05). However, when weight bearing increased to 20% and 30% BW, the subjects’ AHI showed significant differences, decreasing by 0.006 ± 0.004 (*p* = 0.004) and 0.009 ± 0.005 (*p* = 0.001), respectively.

**Table 1:** The static and dynamic arch index of subjects in different weight bearing during walking and gait termination including mean (SD), significance difference and effect size.

		**Weight bearing conditions**				**Pairwise comparison (*p* value)**						**Effect size (_*p*_*η*2)**
		0% BW	10% BW	20% BW	30% BW	0% Vs 10%	0% Vs 20%	0% Vs 30%	10% Vs 20%	10% Vs 30%	20% Vs 30%	
**AHI**	**Static**	0.376 (0.008)	0.373 (0.009)	0.370 (0.008)	0.367 (0.008)	0.680	0.004^*^	0.001^*^	0.035^*^	0.001^*^	0.004^*^	0.690
**AI**	**Walk**	23.70 (1.84)	24.72 (1.98)	25.30 (2.12)	25.66 (2.05)	0.000^*^	0.000^*^	0.000^*^	0.000^*^	0.000^*^	0.397	0.651
	**PGT**	24.49 (2.35)	25.16 (2.55)	25.59 (1.94)	25.79 (2.24)	0.000^*^	0.000^*^	0.000^*^	0.028^*^	0.001^*^	0.332	0.329
	**UGT**	24.44 (2.07)	25.06 (2.17)	25.58 (2.06)	25.94 (1.98)	0.002^*^	0.000^*^	0.000^*^	0.003^*^	0.000^*^	0.007^*^	0.425

**Notes.**

* Significant difference between different weight bearing conditions (*p* < 0.05).

AI data during walking and two types of gait termination under different weight bearing conditions are presented in Table 1, Figures 4B–4D and Data S2. Significant differences were observed except under the loading condition of 20% BW to 30% BW (all *p* < 0.05, *p* = 0.397). No significant differences were observed when weight bearing increased from 20% BW to 30% BW (*p* = 0.332). In addition, significant increases were recorded when adding weight during UGT (all *p* < 0.05).

### Plantar pressure

The means, SDs and results of the statistical analysis of the maximal pressure of the identified eight anatomical regions were determined based on the 180 walking and gait termination trials (twelve subjects × three types × five trials), shown in Figs. 4E–4G and Data S3.

During walking maximal pressure in all anatomical regions revealed an increased trend with added body weight. In M2, the maximal pressures were increased significantly from no weight bearing to increases of 10% and 30% BW (*p* = 0.047, *p* = 0.003). Moreover, significant increases were exhibited in the subjects’ rearfoot. In HM significant maximal pressure increases were observed from zero weight bearing to 20% and 30% BW (*p* = 0.012, *p* = 0.000). Similarly, maximal pressure increased significantly in HL when adding increases of 20% and 30% BW (*p* = 0.002, *p* = 0.000).

Significant maximum pressure increases were found in the forefoot and rearfoot during PGT. However, it was also observed that no significant differences occurred in the midfoot under the four different weight bearing conditions. Both HL and HM showed significant increases in maximum pressure when weight bearing additions ranged from 0% and 10% to 30% BW (*p* = 0.000, *p* = 0.000; *p* = 0.003, *p* = 0.026).

When the subjects performed UGT, significant maximum pressure increases were observed in the lateral forefoot and midfoot with weight bearing increases. The MF region presented significantly increased maximum pressures ranging from 0% to 20% and 30% BW (*p* = 0.015 and 0.00) respectively. However, it was also observed that no significant differences occurred in the rearfoot with the addition of additional body weight.

## Discussion

Our primary findings were that, (i) the arch revealed a flattening trend in static and dynamic conditions when adding additional weight; (ii) maximal pressure showed an increasing trend in all anatomical regions when body loading was increased. In relation to statistically significant differences, the plantar pressure distribution characteristics of the three types of dynamic gait experiments were different. Taken together, these main findings provide a potential biomechanical explanation for the gait termination pattern observed in individuals subjected to additional body weight.

Our findings indicate decreases in AHI as body loading increased, which suggests that additional weight bearing could result in foot deformation. However, no significant differences were found between 10% BW increases and no increase in weight bearing (*p* = 0.068). This finding indicates that low levels of weight bearing produced relatively small loading/deformations in the foot arch. In the stance phase, the body is subjected to excess downward vertical forces when wearing a weighted vest and the foot arch plays an important role in attenuating the resulting shock (Mei et al., 2019). Although osseous elements such as the subtalar joint provide a significant static support for arch stability, the soft tissue of the foot arch would deform in order to prevent the talus from further sinking and tilting inwards (Richie, 2007; Crary, Hollis & Manoli, 2003). Arch index information suggests that the midfoot contact area increased with increase in loads, and that there was no significant difference until the loads reached 30% BW during walking and PGT. This was consistent with the curve between arch height and loading which Bjelopetrovich & Barrios (2016) also observed. They also reported a ceiling effect and that the decline in arch height gradually slows until it stops changing as load increases. The ceiling effect may be related to the deformation mechanism of plantar fascia and plantar ligament of the medial longitudinal arch. However, when the additional load increased to 30% BW in this study, the AI still showed a significant increase during UGT, which might be due to greater plantar loading, especially in the lateral metatarsal and heel during UGT (Cen, Jiang & Gu, 2019). A net braking impulse (braking force—push-off force) needs to be urgently provided during this movement to adapt a new body posture (Bishop et al., 2002). As a result, further lowering of the arch might lead to foot injuries. Prolonged excessive body loading may also lead to repeated excessive arch deformation, resulting in plantar fasciitis and other foot injuries (Richie, 2007; Wearing et al., 2006).

The experimental results of plantar pressure also support the above conclusion. Plantar pressure distribution changes during walking were consistent with the results of Hotfiel et al. (2017). They demonstrated that maximal plantar pressures increased with loaded body weight and the pattern of increase varied over different anatomical regions. However, different from the previous study, the highest relative increase in maximal pressure was found in the midfoot. We observed significant maximum pressure increases in the forefoot and rearfoot regions, especially in the rearfoot. We speculate that this may be related to the experimental footwear and the range of midfoot area definition, i.e., subjects walked with a neutral shoe, and the midfoot was defined as 30–60% length and 0–100% width of the foot in the previous study (Hotfiel et al., 2017). In our study, subjects walked with barefoot and anatomical regions were artificially divided by the Footscan® 7.0 software. Similar plantar pressure results occurred during PGT, and all metatarsal regions and rearfoot parts showed significant increases with increases in weight. Hotfiel et al. (2017) suggested that the heel, i.e., calcaneus, as the anatomical structure that contacts firstly with the ground after the terminal swing phase and is the main reason that the highest values were measured in the rearfoot area. Excessive peak pressures might increase the risk of foot injuries such as stress fractures (Chatzipapas et al., 2008; Zhou & Ugbolue, 2019; Cen et al., 2020). Plantar pressure changes in the forefoot were consistent with distribution shifts from the lateral to the medial metatarsal in patients with flat feet (Arangio & Salathé, 2001). Similarly, the significant increases in the forefoot and rearfoot were recorded during the PGT stage of the experiment. When unexpected and rapid gait ceases, the lower limbs tend to provide more braking force and less push-off force (Bishop et al., 2002; Hase & Stein, 1998). However, this greater loading might be due to the nature of the action, not primarily due to weight bearing conditions (Sparrow & Tirosh, 2005). Hence there were no significant changes in the maximum pressure on the forefoot and rearfoot as the loading increased. However, the significant increase in plantar loading observed in the midfoot area is consistent with the conclusions of the findings related to the arch index.

Weight bearing activities are common in daily life, although they could adversely affect the arches of the feet and lower limbs. At the same time, weight training is accepted globally as a convenient and inexpensive way to exercise. It also improves muscle strength and other physiological functions for various populations (Normandin et al., 2018). However, the damage to the musculoskeletal system caused by weight training should be noted. Some corresponding measures deserve further study. The application of orthotic insoles might be useful to compensate elevated body weight (Hotfiel et al., 2017). Zhao et al. (2018a) and Zhao et al. (2018b) suggested that arch support functional insoles could effectively improve weight-bearing motor patterns and decrease foot pain. Heel lifts by adding arch support have also been demonstrated to reduce the velocity and displacements of the medial-lateral center of pressure (ML-COP), improving stability during walking (Zhang et al., 2017).

Several limitations should be noted in present study. Firstly, we did not measure the joint kinematics of the lower limb. The data of the ankle, knee and hip might provide direct evidence for observing arch alterations under different loading conditions. Further experimental studies should include a multi-segmental foot model that should provide comprehensive analysis. Secondly, the subjects were required to walk barefoot in this study, and the findings may be different from studies where subjects were wearing shoes.

## Conclusions

Although weight-bearing behaviour is unavoidable in daily life, its adverse effects on the musculoskeletal system of the feet should be noted. The results of this study demonstrate that additional body weight could lead to a collapse of the foot arch owing to bearing excess downward vertical forces, especially during unexpected and rapid gait termination. Under higher body loads, the plantar loading showed greater maximal pressure, which would increase the risk of foot injuries. The overall findings from the study combined with morphology-related results, especially the contribution of the foot arch during gait termination, may provide insights for further biomechanical researches, injury prevention and footwear design.

##  Supplemental Information

10.7717/peerj.8998/supp-1 Data S1 Raw data exported from arch height index (AHI) of subjects in different weight bearing applied for data analyses and preparation for the detailed investigation shown in Fig. 3A and Table 1

10.7717/peerj.8998/supp-2 Data S2 Raw data exported from the dynamic arch index (AI) of subjects in different weight bearing during walking and gait termination applied for data analyses and preparation for the detailed investigation shown in Figs. 3B-D

10.7717/peerj.8998/supp-3 Data S3 Raw data exported from the normalized maximal pressure of subjects in different weight bearing during walking and gait termination applied for data analyses and preparation for the detailed investigation shown in Figs. 3E-G

## Additional Information and Declarations

### Data Availability

The following information was supplied regarding data availability: The raw measurements are available in the Supplemental Files.
